# Creation of Crystalline Orientation of Tin(II) Oxide Polycrystals with High Photocatalytic Activity

**DOI:** 10.3390/molecules30132870

**Published:** 2025-07-06

**Authors:** Svetlana A. Kuznetsova, Olga S. Khalipova, Yu-Wen Chen

**Affiliations:** 1 Department of inorganic chemistry, National Research Tomsk State University, 634050 Tomsk, Russia; onm@xf.tsu.ru (S.A.K.); chalipova@mail.ru (O.S.K.); 2 Department of Chemical and Materials Engineering, National Central University, Jhongli 32001, Taiwan

**Keywords:** tin(II) oxide, microwave synthesis, microwave synthesis, crystallographic texture, photocatalysis, destruction of organic dyes

## Abstract

Tin(II) oxide is a promising material for photocatalytic wastewater treatment. However, the established relationships between particle size, shape, and photocatalytic activity of SnO are contradictory, indicating the influence of other factors. In this work, the effect of the SnO crystallographic texture on its band gap and photocatalytic activity was shown for the first time. The relationship between the methods (microwave and hydrothermal microwave) and synthesis conditions (time, pressure, and chemical composition of the suspension) of polycrystalline tin oxide(II) and the crystallographic texture was studied. The crystallographic texture was estimated by the Harris method using the repeatability factor and the Lotgering coefficient. The formation of crystallites oriented in the growth plane (*00l*) was facilitated by the carbonate medium of the suspension. In the ammonia medium, crystallites were preferably formed in the plane (*h0l*). Increasing the time and pressure leads to the recrystallization of SnO. The band gap energy of the SnO increases from 3.0 to 3.6 eV, and the rate of photodestruction of methyl orange decreases with the growth of crystallites in the (*00l*) plane from 17 to 40%.

## 1. Introduction

Tin(II) oxide is considered a promising material in batteries [1,2,3,4,5], sensors [6,7,8,9,10], and photocatalysts [11,12,13,14] due to its layered tetragonal structure and adjustable defectivity. Polycrystalline materials based on tin(II) oxide exhibit photocatalytic activity in the photodegradation of organic pollutants in water under the influence of ultraviolet light irradiation [15].

Many researchers have reported on the influence of surface morphology, SnO crystallite size, and specific surface area on the optical band gap and photodestruction rate of organic dyes. The values of the band gap (ΔE) and specific surface area (S) are presented in Table 1. In the literature [16], it was established that even a small change in the size of spherical tin oxide crystallites from 22.6 to 19.3 nm (the size was determined by transmission microscopy) led to an increase in specific surface area from 73.46 to 157.45 m^2^/g, and in the optical band gap from 3.70 to 4.20 eV. A similar trend was observed for tin oxide with a flower-like morphology, where band gap energy increased with decreasing particle size and specific surface area increased (7.59 m^2^/g (2.59 eV) [12]; 52.90 m^2^/g (2.65 eV) [17]). However, the particle size of tin oxide differs dramatically with such a slight change in the width of the optical band gap. It should also be noted that the ΔE determined by different authors for SnO crystallites of different sizes was characterized by close values (2.75 eV (20–30 nm) [18], 2.65 eV (10–30 nm) [17], and 2.59 eV (800 nm) [12]).

A comparison of the X-ray diffraction patterns of tin(II) oxide presented in the works [12,17,18] reveals significant variations in the intensity of diffraction maxima corresponding to the same planes. This indicates the difference in the crystalline structure of SnO polycrystals, including those with similar morphology, which is determined by the anisotropy of the growth rate of SnO crystallites in different planes [21,22], depending on the chemical composition of the precursor and the environment.

**Figure 1:**
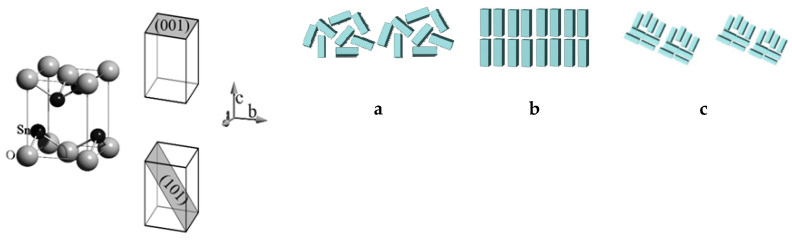
Texture-less state (**a**), single component (**b**), and multi-component texture (**c**).

In addition, theoretical calculations based on various models using density functional theory have shown different sorption and optical properties for different faces of oxide crystallites [23,24,25,26,27]. The experimental studies of polycrystalline materials based on titanium oxide (anatase) with the tetragonal syngony have also supported these findings [28]. Thus, the properties of tin(II) oxide, which has a tetragonal layered structure and is characterized by anisotropy in crystallite growth, may depend not only on the size and shape of the building blocks and agglomerates but also on the texture components of the polycrystalline powder (Figure 1). Such studies for tin(II) oxide are absent in the literature.

Among the various methods for producing polycrystalline tin monoxide, thermal treatment of colloidal solutions of tin(II) oxohydroxide stands out [8,16,29,30,31,32,33,34,35,36,37,38,39,40,41,42,43,44,45]. This method allows for controlling the oxidation process of tin(II) by using a dispersion medium with different compositions [46], as well as using different types of heating to prevent local overheating of the reaction mixture. Tin(II) chloride and soluble bases in aqueous or alcoholic media are mainly used as precursors. In order to suppress the hydrolysis of the salt, solutions are acidified. Alkali metal hydroxides or ammonia solutions are mainly used as bases [31,47,48,49]. Using these precipitants causes the nucleation rate to exceed the crystallite growth rate (rapid precipitation [50]). In case of rapid precipitation, the reaction occurs under non-equilibrium conditions and is difficult to control. The use of compounds such as sodium carbonate, hexamethylenetetramine, urea, etc. [51] as precipitants allows for a reduction in the rate of nucleation. These compounds do not contain hydroxyl groups, but they are formed during their hydrolysis process, which occurs slowly. In the case of slow precipitation, nanosized oxide particles are formed mainly due to a decrease in local concentration gradients and the degree of supersaturation of the solution. The use of solid inorganic tin(II) salts as a source of Sn^2+^ ions in an aqueous or aqueous–alcoholic solution has some disadvantages, such as low and long-term solubility of the salt and a high capacity for hydrolysis. In this regard, the salt can be obtained by dissolving metallic tin in excess of concentrated acids, such as hydrochloric acid. Then, tin(II) oxohydroxide can be precipitated from the acidic Sn^2+^ solution. The use of the method for obtaining SnO polycrystals from tin(II) oxyhydroxide suspension is of interest as it allows for the production of SnO crystals with different crystallographic textures, since changing the composition of the dispersion medium makes it possible to control the growth of crystallites in different planes.

Based on the results of previous studies, it has been found that controlling the morphology of SnO polycrystals is important because the samples with different shapes and sizes exhibit different properties. However, knowledge of the shape and size of crystallites in a polycrystal is not sufficient to explain their properties. It is also necessary to understand the effect of crystallite orientation in polycrystalline SnO powders on their properties, as well as the possibility of creating conditions for the synthesis of tin(II) oxide with a specific crystallographic texture. This study was devoted to obtaining optically active polycrystalline SnO powders with different crystallographic textures and to establishing the effect of the preparation conditions from a tin(II) oxyhydroxide suspension on the crystallographic texture of the oxide polycrystals and their photocatalytic properties.

## 2. Results and Discussion

### 2.1. Preparation of Polycrystalline SnO from Sn_6_O_4_(OH)_4_ Suspensions by Microwave and Hydrothermal–Microwave Treatments

**Figure 2:**
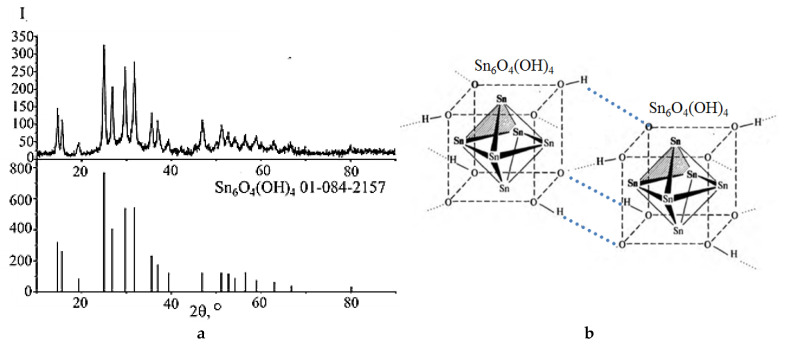
XRD pattern of the dispersed phase of Sn_6_O_4_(OH)_4_ suspensions (**a**) and the structure diagram of two protonated [Sn_6_O_8_] clusters (**b**).

According to the XRD results (Figure 2a), the dispersed phase in all suspensions was tin(II) oxohydroxide, which has a composition of Sn_6_O_4_(OH)_4_ and crystallizes in a tetragonal structure with lattice parameters a = b = 7.96 Å; c = 9.14 Å. In the Sn_6_O_4_(OH)_4_ structure, in addition to Sn–O bonds, there are also Sn−Sn bonds, which form pseudocubic [Sn_6_O_8_] clusters, linked together by hydrogen bonds into infinite structures (Figure 2b). The boiling of Sn_6_O_4_(OH)_4_ aqueous suspension for 80 min under conventional heating, as well as keeping it in a microwave oven for 15 min, does not lead to the decomposition of tin(II) oxyhydroxide. The process of SnO formation is initiated by the OH^−^ ion (alkaline environment).

**Figure 3:**
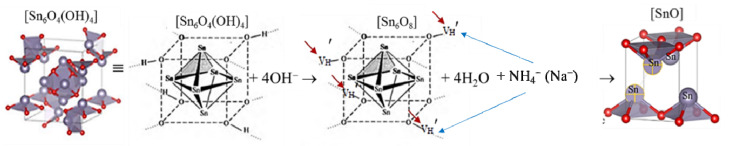
Reaction equations and scheme of SnO formation from Sn_6_O_4_(OH)_4_ in an alkaline environment.

The Sn_6_O_4_(OH)_4_ is stable in an ammonia environment up to a pH of 10.5 and 80 °C. At higher temperatures, when the suspension boiled, the formation of a true solution was also not observed. Therefore, the process of producing tin(II) oxide from tin(II) oxohydroxide in an alkaline environment at the stage of dissolution of the oxohydroxide, with the concomitant formation of an anionic hydroxocomplex in solution, is questionable. The reaction most likely occurred at the phase boundary and the dispersion environment/dispersed phase. The formation of the double electric layer occurs with the participation of hydroxyl ions of the medium in the reaction with solid Sn_6_O_4_(OH)_4_. The water molecules formed in this case are removed into the solution. This leads to the formation of electron defects on the oxygen of the [Sn_6_O_8_] framework with subsequent rearrangement of its structures into an oxide (SnO) due to the diffusion of coordinative unsaturated lattice oxygen and its interaction with electron defects. The reactions and the scheme of SnO formation from Sn_6_O_4_(OH)_4_ in an alkaline environment are shown in Figure 3.

Sn_6_O_4_(OH)_4_ + 4(NH_3_·H_2_O) = (NH_4_)_4_Sn_6_O_8_ + 4H_2_O (1)

(NH_4_)_4_Sn_6_O_8_ = 6SnO + 4NH_3_ + 2H_2_O (2)

### 2.2. Phase Composition, Structure, Crystallographic Texture, and Surface Morphology of Polycrystalline SnO

**Figure 4:**
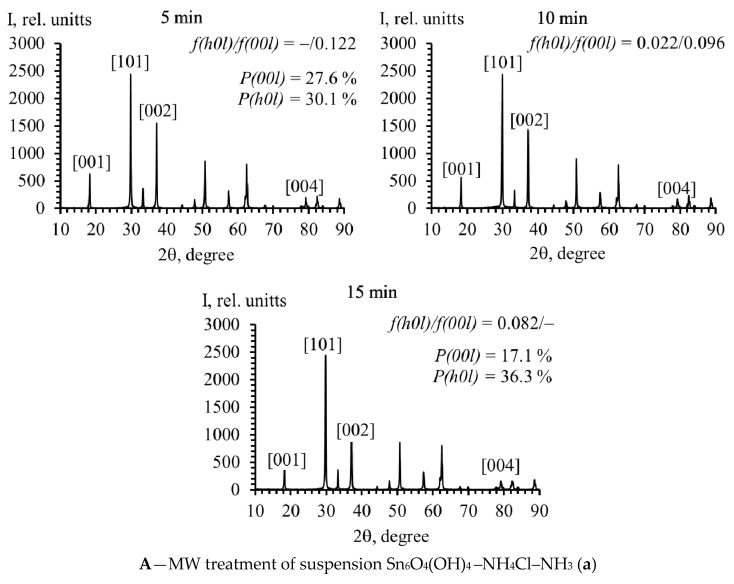

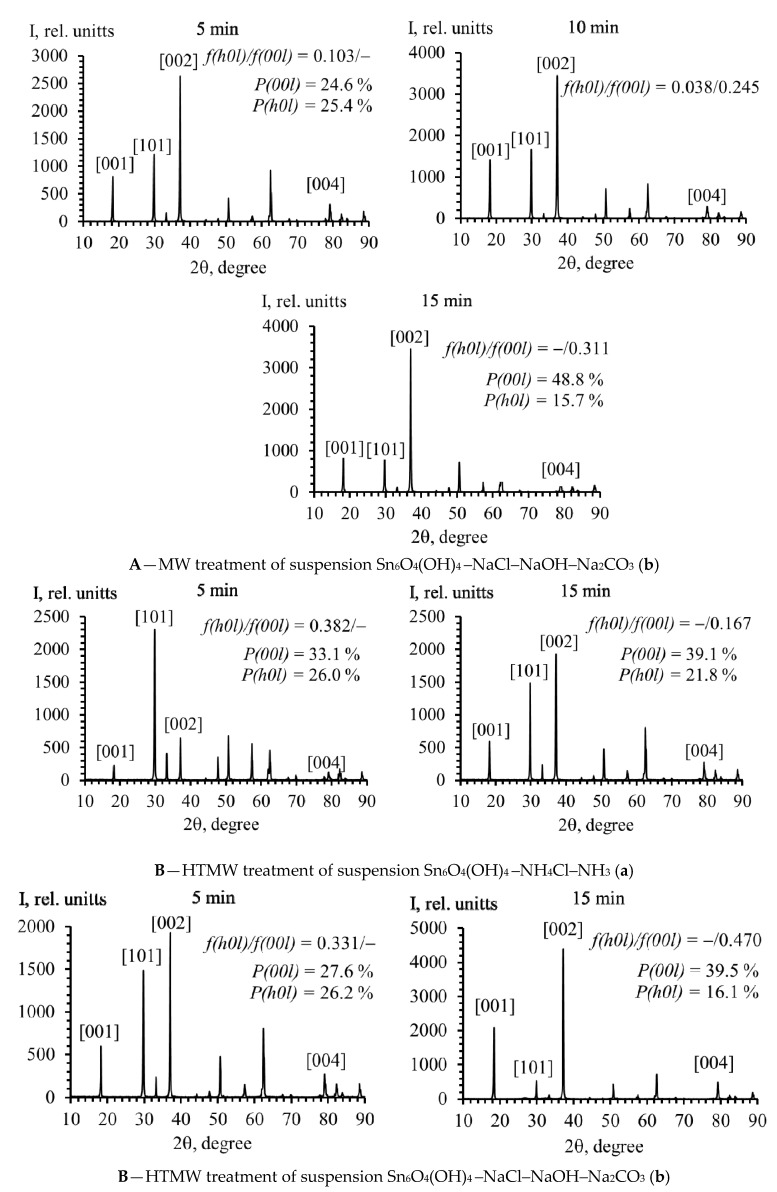
XRD patterns of SnO powders obtained by heat treatment of Sn_6_O_4_(OH)_4_ suspensions with different medium compositions and the values of the repeatability (p_(*hkl*)_ ± 0.2%) and Lotgering (*f*_(*hkl*)_ ± 0.02%) factors.

Tin(II) oxide was formed from tin(II) oxohydroxide suspensions with different dispersion medium compositions as a result of conventional, MW, or HTMW heating during 5 min. In all cases, the tetragonal structure of SnO is characterized by the same values of the crystal lattice parameters a = b = 3.80 Å; c = 4.84 Å; however, the ratio of the intensities of the diffraction maxima is different (Figure 4). This indicates different crystallographic textures of the samples. The repeatability factor (P_(*hkl*)_), calculated by the Harris method, for SnO crystallites oriented in the (*00l*) planes (e.g., (*001*), (*002*), (*004*)) and the (*h0l*) planes (e.g., (*101*), (*202*)) was about 27.6% and 30.1%, respectively, for samples obtained by the microwave treatment of a suspension in an ammonia medium for 5 min (Figure 4(Aa)).

For samples prepared in a sodium carbonate medium, the corresponding values are 24.6% and 25.4% (Figure 4(Ab)). The confidence interval of the repeatability factor was ±0.2%. It can be concluded from this that a slight preference for the orientation of tin monoxide crystallites oriented in the (*00l*) plane is observed in an ammonia medium.

As can be seen from Figure 4(Ba,Bb), the preference for crystallite growth in this direction is also observed for the oxide prepared by HTMW treatment of an ammonia suspension of Sn_6_O_4_(OH)_4_.

It has been determined that subjecting the obtained SnO suspensions to MW and HTMW heating for 15 min resulted in a change to the crystallographic texture. In an ammonia medium, the SnO recrystallization under microwave conditions results in an increase in crystallites oriented along the (*h0l*) plane (P_(*h0l*)_ = 36.3%, P_(*00l*)_ = 17.1%). In a carbonate environment, this process leads to the orientation of crystallites in the (*00l*) plane (P_(*00l*)_ = 48.8%, P*_(h0l)_* = 15.7%). This also follows from the values of the Lotgering factor, which increase for the (*00l*) plane in the case of using a carbonate suspension (5 min – *f*_(*h0l*)_/*f*_(*00l*)_ = 0.103/–; 15 min – *f*_(*h0l*)_/*f*_(*00l*)_ = –/0.311), and decrease for an ammonia suspension (5 min – *f*_(*h0l*)_/*f*_(*00l*)_ = –/0.122; 15 min – *f*_(*h0l*)_/*f*_(*00l*)_ = 0.082/–). The increase in the time of keeping tin monoxide in the suspension under HTMW, regardless of the composition of the dispersion medium (ammonia or carbonate), leads to the increase in crystallites oriented in the (*00l*) plane.

**Figure 5:**
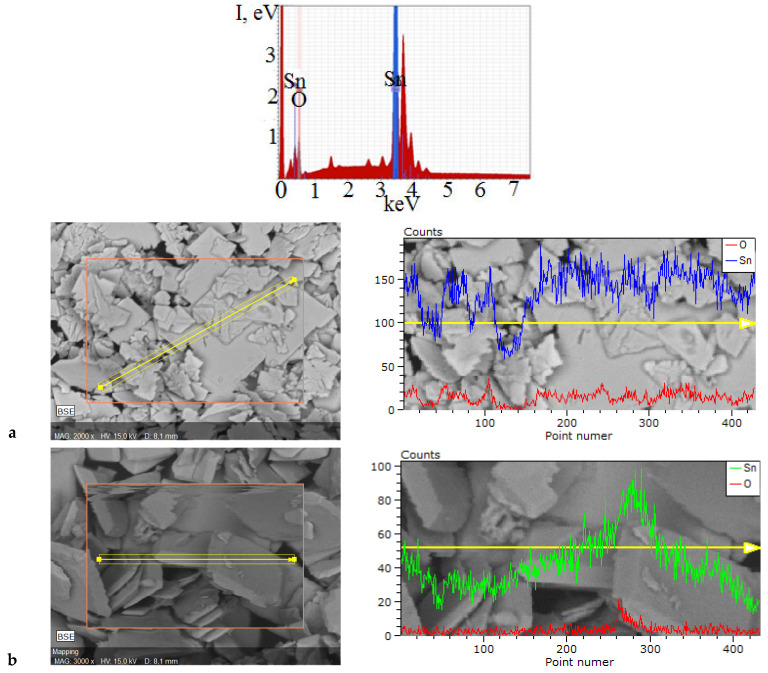
X-ray energy dispersive spectrum and point maps of the distribution of elements on the surface of SnO samples obtained by MW treatment of ammonia (**a**) and carbonate (**b**) suspensions during 15 min.

The results of XRD analysis of the decomposition products of Sn_6_O_4_(OH)_4_ under MW irradiation are consistent with the results of qualitative and quantitative EDX with SEM analysis (Figure 5).

Qualitative EDX results indicate that all samples contain only Sn and O elements. According to the distribution maps of these elements on the surface of the samples, it can be concluded that Sn and O are distributed uniformly. This confirms the presence of the phase of SnO. Quantitative EDX results confirm the formation of a single-phase powder by microwave treatment of suspensions at 5, 7, 10, and 15 min. The atomic percentage ratio of the elements Sn:O is 1:1 at each point of these samples (Table 2).

**Figure 6:**
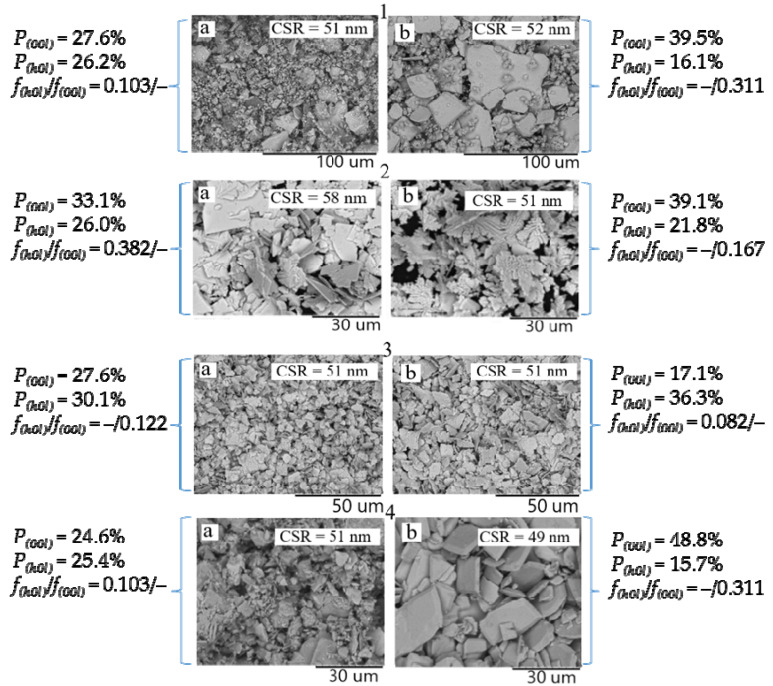
Microstructure of SnO powders obtained by HTMW treatment of the carbonate suspension (**1**) for 5 min (**a**) and 15 min (**b**); of the ammonia suspensions (**2**) for 5 min (**a**) and 15 min (**b**); and by MW treatment of the ammonia suspension (**3**) for 5 min (**a**) and 15 min (**b**); of the carbonate suspension (**4**) for 5 min (**a**) and 15 min (**b**).

The SEM results of polycrystalline SnO powders are shown in Figure 6.

As can be seen in Figure 6, the SnO polycrystals obtained from the same precursor have similar surface morphology. All SnO powders obtained from suspensions with ammonia are polygonal plates with a length of 5 to 20 μm and a thickness of not more than 0.5 μm. As the microwave treatment time increases, the particles become larger, indicating agglomeration. The surface morphology of SnO samples obtained from the carbonate suspension is characterized by the presence of particles with larger sizes. They become more regular in shape (rectangular and square) and thicker (more layered). The thickness of these particles can reach up to 4 μm, and their length can be up to 56 μm.

Polycrystalline SnO samples obtained by hydrothermal–microwave treatment of the carbonate suspension Sn_6_O_4_(OH)_4_ for 5, 10, 15 min differ in surface morphology. As can be seen in Figure 6, the SnO samples are a mixture of oxide particles with different sizes and shapes. The appearance of the SnO agglomerates obtained in 5 min by HTMW treatment of the carbonate suspension is similar to that of the SnO agglomerates obtained from ammonia suspensions. The only difference is their tendency to stick together (stratification) and the presence of a large number of square and rectangular particles. The increase in HTMW treatment time leads to the agglomeration of oxide particles, resulting in an increase in their size and thickness. A comparison of the surface morphology of the samples indicates that there is a critical size of the agglomerate, which is destroyed under pressure over time. This nature of the change in the oxide surface morphology over time indicates a recrystallization process, which is confirmed by the values of the Lotgering factor.

The appearance of SnO agglomerates obtained from different suspensions is consistent with the assumption that the predominance of crystallite growth faces in the (*00l*) plane, characteristic of SnO crystallites formed in a carbonate suspension, leads to the formation of more layered and geometrically “regular” SnO polycrystals, in agreement with the results of the work [52].

All the tin monoxide polycrystals were resistant to water and light.

### 2.3. Surface Properties of Polycrystalline SnO

**Figure 7:**
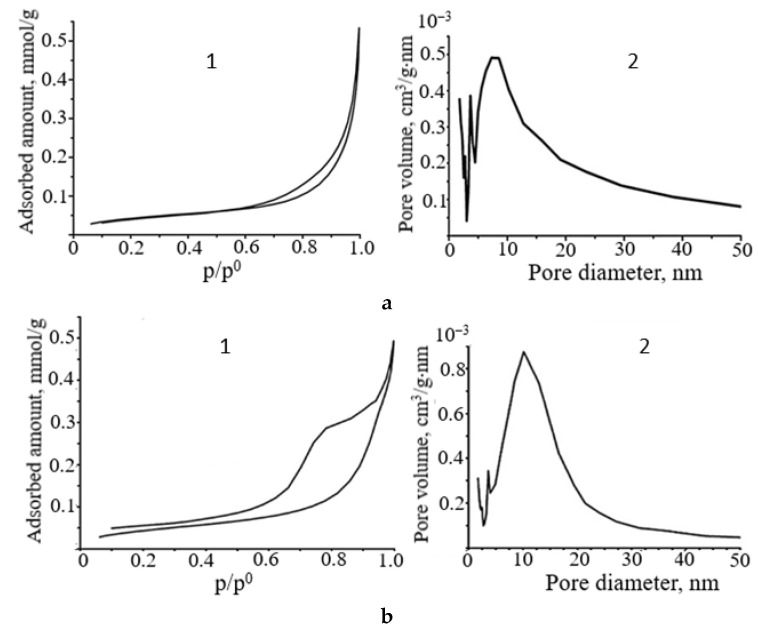
Nitrogen adsorption–desorption isotherm (**1**) and pore size distribution curve (**2**) of SnO obtained by microwave treatment of the ammonia Sn_6_O_4_(OH)_4_ suspension for: (**a**)—5 min (P_(*00l*)_ = 27.6%, P_(*h0l*)_ = 30.1%); (**b**)—15 min (P_(*00l*)_ = 17.1%, P_(*h0l*)_ = 36.3%).

According to the results of nitrogen sorption (BET method), the SnO samples obtained from the ammonia suspension of Sn_6_O_4_(OH)_4_ at MW treatment for 5–15 min are characterized by a low specific surface area of 3.0–3.5 m^2^/g with an average pore size of around 8 nm. Based on the shape and position of the hysteresis loop on the adsorption–desorption curves of the samples obtained under microwave conditions, the SnO powders can be classified as H3-type adsorbents with slit-shaped pores, according to the IUPAC classification (Figure 7). An analysis of the pore size distribution curves shows that the SnO powders exhibit a mesoporous structure with micropore impurities. The SnO sample prepared by microwave treatment for 5 min is characterized by a narrow hysteresis loop (Figure 7(a1)). As demonstrated in Figure 7(b1), the maximum pore volume of these polycrystalline oxides was 4.8 × 10^−4^ cm^3^/g·nm with a diameter of 7–9 nm.

Increasing the microwave irradiation time up to 7 min did not lead to a change in the adsorption–desorption isotherm curve of the sample. However, there is a slight decrease in the pore volume to 3 × 10^−4^ cm^3^/g·nm with a diameter of 4–6 nm and to 1.2 × 10^−4^ cm^3^/g·nm with a diameter of 7–9 nm. The SnO sample synthesized within 15 min is a mesoporous adsorbent with a more heterogeneous pore shape. This sample is characterized by a wider hysteresis loop area on the adsorption–desorption curve, a larger pore volume of up to 8 × 10^−4^ cm^3^/g·nm, and a wide distribution of pore diameters from 5 nm to 20 nm. A slight enhancement of the pore volumes of polycrystalline SnO with increasing MW irradiation time can be attributed to the formation of a larger number of oxide crystallites oriented in the (*h0l*) plane. In view of the fact that SnO is already formed after 5 min of treatment, the change in its texture over time can only be related to the recrystallization process.

**Figure 8:**
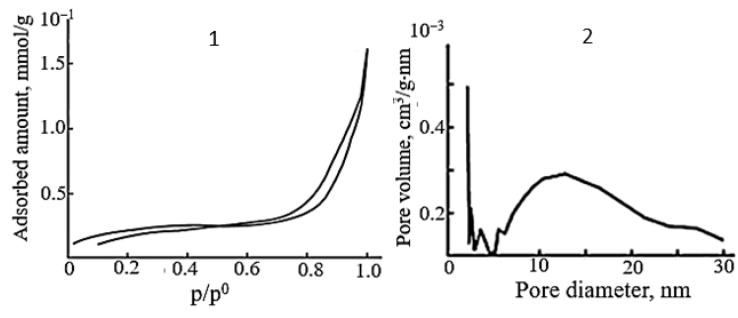
Nitrogen adsorption–desorption isotherms (**1**) and pore size distribution curve (**2**) of SnO obtained by microwave treatment of carbonate Sn_6_O_4_(OH)_4_ suspension for 15 min (P_(*00l*)_ = 48.8%, P_(*h0l*)_ = 15.7%).

The low-temperature nitrogen adsorption results (Figure 8) indicate that the surface area of the SnO sample prepared by MW irradiation of a carbonate Sn_6_O_4_(OH)_4_ suspension is 0.4 m^2^/g, which is an order of magnitude smaller than the surface area of SnO samples obtained under the same conditions from the ammonia suspension.

The SnO powder prepared from the carbonate suspension has a mesoporous structure with micropore impurities. However, this sample is characterized by a narrower hysteresis loop area and a smaller pore volume of 2 × 10^−4^ cm^3^/g·nm. Consequently, an increase in the number of crystallites grown in the (*00l*) plane of the polycrystalline SnO powder leads to a greater degree of agglomeration (layer fusion) and a decrease in the pore volume. The change in the pore size and volume results in a change in the sorption properties of the SnO samples.

**Figure 9:**
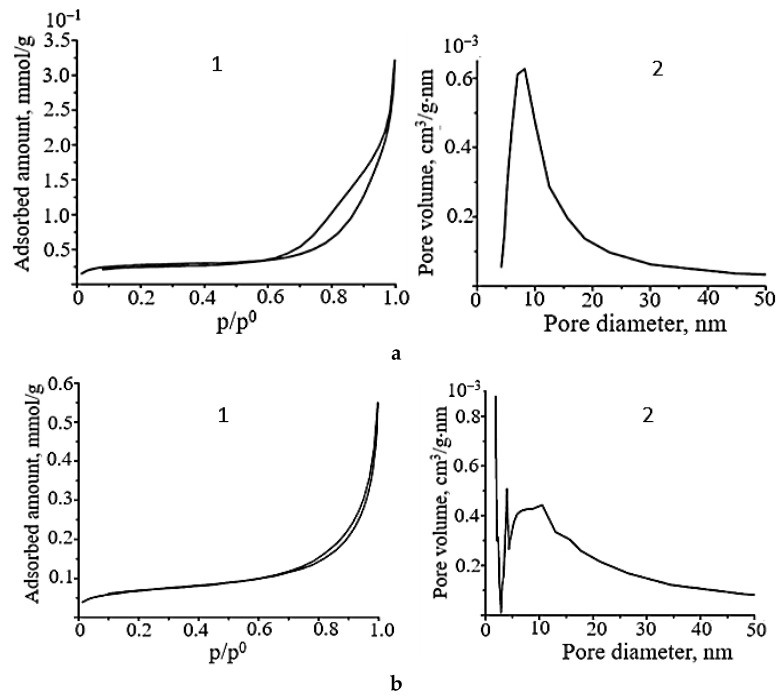
Nitrogen adsorption–desorption isotherm (**1**) and pore size distribution curve (**2**) of SnO obtained by HTMW treatment of ammonia Sn_6_O_4_(OH)_4_ suspension for (**a**): 5 min (P_(*00l*)_= 33.1%, P_(*h0l*)_ = 26.0%); (**b**): 15 min (P_(*00l*)_ = 39.1%, P_(*h0l*)_ = 21.8%).

**Figure 10:**
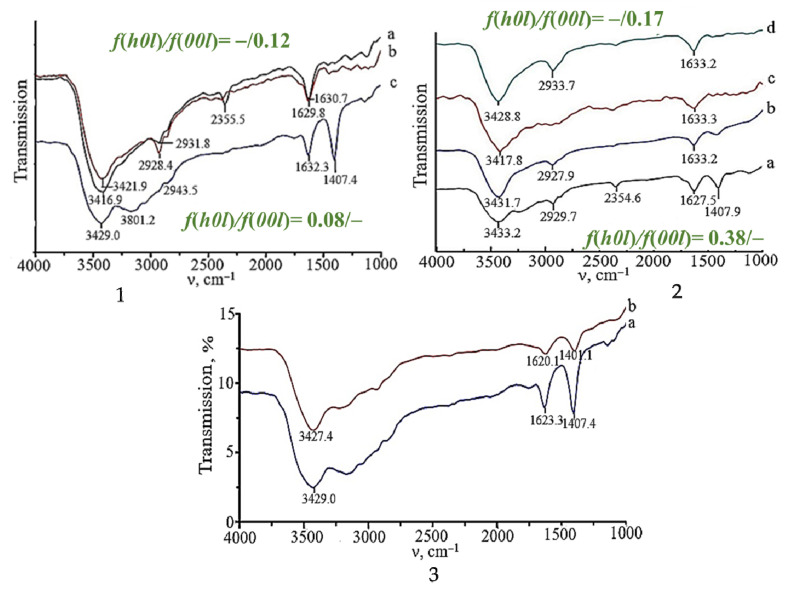
IR spectra of polycrystalline SnO obtained from ammonia Sn_6_O_4_(OH)_4_ suspension by MW treatment (**1**) for a: 5 min; b: 7 min; and c: 15 min; HTMW treatment (**2**) for a: 5 min; b: 7 min; c: 10 min; and d: 15 min; by MW treatment for 15 min (**3**): a: before holding in vacuum, b: after holding in vacuum.

The adsorption–desorption isotherms and pore size distribution curves of SnO samples prepared from the ammonia suspension under hydrothermal–microwave conditions are shown in Figure 9. According to the shape of the hysteresis loops, all SnO powders are mesoporous adsorbents and characterized by low values of specific surface area of 2–5 m^2^/g and average pore size of 14 nm. The pore volume is (4–6) × 10^−4^ cm^3^/g·nm with a diameter of 5–20 nm. Microporosity appears in the tin(II) oxide samples when the HTMW treatment time is increased from 5 to 15 min. Therefore, as in the case of microwave-exposed samples, the pore volume slightly decreases with an increase in the number of crystallites oriented in the (*00l*) plane in the polycrystals. Despite the close value of the pore volume and specific surface area of tin monoxide samples with different crystallographic textures, the hydroxyl and hydrate layer on their surface is different. An analysis of the IR spectra of SnO powders shows that the surface of all tin monoxide samples contains molecules of adsorbed water. This is evidenced by absorption bands in the region from 3450 to 2360 cm^−1^ (stretching vibrations of hydroxyl groups [53]) and from 1650 to 1410 cm^−1^ (deformation vibrations of water molecules [54]) (Figure 10(1,2)). Evidence that all the listed bond vibrations correspond to physically adsorbed water molecules is the change in their intensity until they completely disappear after vacuum treatment of the SnO samples (Figure 10(3)).

The increase in the number of crystallites oriented in the (*00l*) plane in polycrystals (the value of the Lontgering factor in the (*00l*) plane increases) leads to a decrease in the amount of adsorbed water molecules. As can be seen from Figure 10(1,2), the amount and intensity of absorption bands related to the vibrations of the bonds of adsorbed water molecules on the SnO surface decrease.

**Figure 11:**
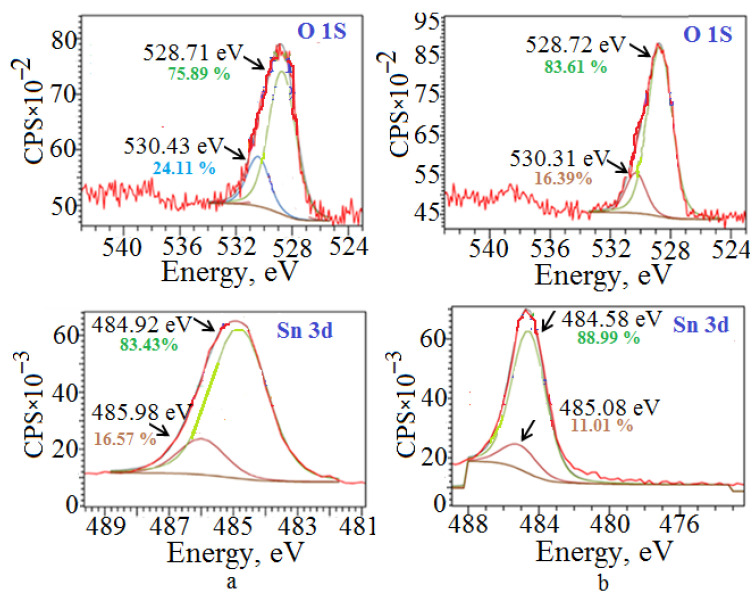
XPS of SnO powders with a repeatability factor of crystallites oriented in the (*00l*) plane of (**a**) 17.1% and (**b**) 48.8%.

Different amounts of coordinative unsaturated tin and oxygen sites in the samples are confirmed by the results of XPS spectroscopy (Figure 11).

The scanning spectra indicate the presence of characteristic peaks for the Sn 3d_3/2_, Sn 3d_5/2_, and O1s states in all SnO samples, which corresponds to the presence of a bond between Sn^2+^ and O^2−^ in the tetragonal lattice [53,55]. The maxima at 484.6 eV; 484.8 eV in the XPS spectra of Sn 3d_3/2_, Sn 3d_5/2_ indicate the presence of a bond between tin(II) and oxygen in the [SnO_4_] lattice, and the maxima in the region of 485–486 eV confirm the presence of electron-deficient tin cations in the lattice. There are two maxima in the O 1s XPS spectra: a maximum at 528.7 eV, characterizing the energy of the oxygen-tin bond in the [SnO_4_] lattice, and a maximum at 530.43 eV, characterizing the energy of the Sn bond with the oxygen of the adsorbed water.

However, in SnO samples, the texture of which is distinguished by the lower number of crystallites in the (*00l*) plane (sample P_(*00l*)_ = 17.1%, Figure 11a), there is a greater number of electron-deficient tin cations of 16.57% (peak at 485.98 eV) and a greater amount of adsorbed water of 24.11% (peak at 530.43 eV). For the sample with P_(*00l*)_ = 48.8%, the electron-deficient tin cations (Figure 11b) are 11.01% (peak at 485.08 eV), and the amount of adsorbed water is 16.39% (peak at 530.31 eV).

### 2.4. Photocatalytic Properties of Polycrystalline SnO with Different Crystallographic Textures

The optical band gap (ΔE) and photocatalytic properties of polycrystalline SnO powders with different crystallographic textures were studied. Tin(II) oxide powders that are texture-less with respect to crystallites oriented in the growth plane (*00l*) (*f*_(*00l*)_ = –, *f*_(*h0l*)_ = 0.08–0.38) were assigned to group A. Group B included polycrystalline SnO powders with a crystallographic texture in the growth plane (*00l*) (*f*_(*h0l*)_ = –, *f*_(*00l*)_ = 0.12–0.47). The values of the repeatability factors (P_(*00l*)_), the Lotgering coefficients (*f*_(*h0l*)_ and *f*_(*00l*)_), and the designations of the SnO powders belonging to different groups are given in Table 3.

**Figure 12:**
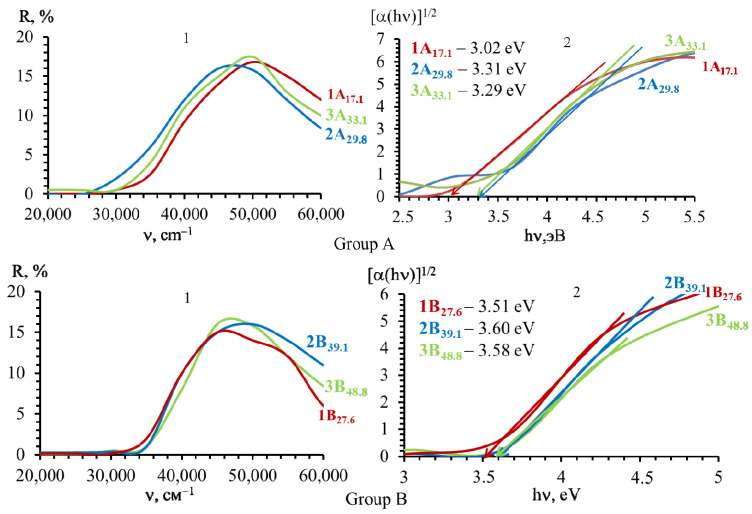
(**1**) Diffuse reflectance spectra and (**2**) Tauc plots for the estimation of direct band gap of polycrystalline SnO powders that are texture-less (Group A) and with a crystallographic texture (Group B) respect to crystallites oriented in the growth plane (*00l*).

Based on the diffuse reflectance spectra, the position of the edge of the intrinsic absorption of the studied SnO powders was analyzed, and the values of the band gap energy were calculated (Figure 12).

The experimental results of the optical band gaps for all polycrystalline SnO powders are in the range from 3.0 to 3.6 eV, which is in agreement with the literature [2,11]. The comparison of the band gaps shows that the polycrystalline tin(II) oxide powders of Group A are characterized by lower values (ΔE = (3.02–3.31) ± 0.02 eV) than the samples of Group B (ΔE = (3.51–3.60) ± 0.02 eV).

**Figure 13:**
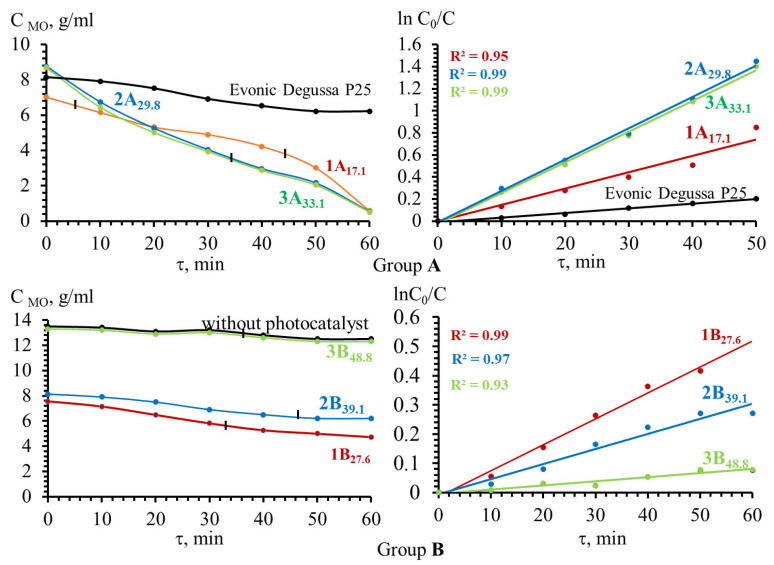
Kinetic curves of the photocatalytic degradation of MO under the influence of UV irradiation (λ = 342 nm) without SnO powder and in the presence of SnO powders that are texture-less (Group A) and with a crystallographic texture (Group B) respect to crystallites oriented in the growth plane (*00l*) and Evonic Degussa P25.

The conducted studies of sorption of methyl orange and photocatalytic activity of prepared SnO powders in the model reaction of the organic azo degradation allowed to establish that the photocatalytic properties of polycrystalline powders depend on the number of crystallites oriented in the plane (*00l*). As can be seen in Figure 13, all SnO powders of group A are photocatalytically active. The degree of MO conversion for 60 min under UV light irradiation was 95–96%.

The SnO polycrystals from group B exhibit photocatalytic properties only those that contain less than 40% of crystallites oriented in the (*00l*) plane. These are polycrystalline SnO powders 1B_27.6_ and 2B_39.1_, which have a repeatability factor of P_(*00l*)_ = 27.6% and P_(*00l*)_ = 39.1%, respectively. The degree of MO conversion for 60 min of UV irradiation is 65.0 wt. % and 54.1 wt. % for 1B_0.28_ and 2B_0.39_ samples, respectively (Table 4). Polycrystalline SnO powders characterized by a repeatability factor P_(*00l*)_ = 48.8% (3B_48.8_) do not possess photocatalytic properties in relation to methyl orange.

Polycrystalline SnO powders characterized by a repeatability factor P_(*00l*)_ = 48.8% (3B_48.8_) did not possess photocatalytic activity for methyl orange destruction.

## 3. Materials and Methods

Tin(II) oxide was synthesized by thermal, microwave, and hydrothermal–microwave treatments of Sn_6_O_4_(OH)_4_ suspension. The suspensions of tin(II) oxohydroxide were obtained in two stages. First, 4 g of metallic tin (Special Metallurgy, Yekaterinburg, Russia) was dissolved in 75 mL of concentrated hydrochloric acid (CP grade, Base No. 1 Chemicals, Staraya Kupavna, Russia) to produce a tin(II) chloride solution. At the second step, 75 mL of the tin(II) chloride hydrochloric acid solution was mixed with ammonia solution (25 mas. %, chemically pure, Base No. 1 Chemicals, Staraya Kupavna, Russia) or sodium carbonate (chemically pure, Base No. 1 Chemicals, Staraya Kupavna, Russia) until the pH of the suspension was from 8 to 10. These suspensions consisted of Sn_6_O_4_(OH)_4_ particles dispersed in different media: H_2_O, NH_4_Cl (in NH_3_ precipitant); NaOH, NaCl, NaHCO_3_, Na_2_CO_3_, and H_2_O (in Na_2_CO_3_ precipitant). The reactions occurring during the preparation of these suspensions can be represented by the following chemical equations:

with NH_3_

NH_3_ + H_2_O ⇆ NH_4_^+^ + OH^−^ (3)

NH_3_ + HCl ⇆ NH_4_Cl (4)

6SnCl_2_ +12NH_3_ + 8H_2_O = Sn_6_O_4_(OH)_4_↓ + 12NH_4_Cl (5)

with Na_2_CO_3_

Na_2_CO_3_ + 2HCl = 2NaCl + H_2_O + CO_2_ (6)

Na_2_CO_3_ + H_2_O ⇆ NaHCO_3_ + NaOH (7)

NaOH + HCl = NaCl + H_2_O (8)

6SnCl_2_ + 12NaOH = Sn_6_O_4_(OH)_4_↓ + 12NaCl + 4H_2_O (9)

The as-prepared suspensions were then exposed to microwave or hydrothermal–microwave treatments. Microwave (MW) treatments of suspensions were carried out in a microwave oven (2450 MHz, made in China) with a radiation power of 539 W for 3, 5, 10, and 15 min. A 200 mL suspension of tin(II) oxohydroxide was placed in a 400 mL heat-resistant glass beaker. The temperature in the reaction mixture was controlled by a mercury thermometer during the interruption of the microwave action on the suspension. The temperature corresponded to 110–120 °C. The experiment was repeated 10 times to confirm the result. The hydrothermal–microwave treatment (HMWT) of the suspension was carried out in a 100 mL Teflon autoclave. The autoclave was filled with 50% (50 mL) of suspension and placed in an MS-6 Volta unit. The power of microwave radiation (2450 MHz) was 539 W. The rate of pressure rise in the autoclave was 0.033 atm/s. The pressure was monitored using an optical sensor, and pressure changes were recorded using software built in the microwave MS-6 Volta (Volta, Saint-Petersburg, Russia). The hydrothermal–microwave treatment time for the suspensions was 3, 5, 10, and 15 min. The experiment was repeated 6 times to confirm the result. For comparison, an aqueous solution of tin(II) oxohydroxide was heat-treated by boiling on a hot plate for 80 min.

As a result of the heat treatment of suspensions, tin(II) oxohydroxide was decomposed and tin(II) oxide was formed:

Sn_6_O_4_(OH)_4_ = 6SnO +2H_2_O (10)

The obtained SnO samples were separated by centrifugation and washed with distilled water until the pH reached 7 and dried at 90 °C.

The phase composition, structure of polycrystalline oxides, and the precursor for their synthesis were studied using X-ray differentiation (XRD) analysis. XRD patterns were collected using a Rigaku Miniflex 600 diffractometer (Rigaku, Tokyo, Japan) with Cu_Kα_ radiation in the range of 2–90° (2θ) with a step of 0.02° and a scanning speed of 2 deg/min. The phase composition of the samples was established using the PDF-4 database and the POWDER CELL 2.4 full-profile analysis program (PDF-2 Release 2012, PD2C120726-2491). The crystallite size (CSR coherent scattering region) was calculated using the Scherrer equation: $D_{XRD}=\frac{K\lambda }{\beta \cdot \cos\theta }$, where D_XRD_ is the crystallite size; K is the dimensionless shape factor with a value close to unity; λ is the wavelength of X-ray radiation; and β—the line broadening at half maximum intensity. The error in calculating the coherent scattering regions was ±4%. The Rietveld method was used to refine the structure of polycrystalline oxides using the ReX powder diffraction program (ReX 0.9.4 version). The texture was taken into account in the full-profile refinement using the March–Dollas function.

The degree of crystal orientation in polycrystalline oxides was assessed by measuring the intensities of diffraction peaks in the XRD patterns of the samples and calculating the Lotgering coefficients (*f*) and the repeatability factor (P_(*hkl*)_) [56]: $f=\frac{P-P_{0}}{1-P_{0}}$; $P=\frac{\sum I(I00)}{\sum I(\mathrm{hkl})}$; $P_{0}=\frac{\sum I_{0}(I00)}{\sum I_{0}(\mathrm{hkl})}$, where *I* and *I*_0_ are the relative intensities of each main reflection peak on the (*hkl*) plane in the XRD pattern of the studied samples and the texture less sample, respectively, *P* and *P*_0_ are the calculated values of the repeatability factor for XRD patterns in the diffraction range (2θ) from 10° to 90°. All studies were performed on a single diffractometer with a Cu_Kα_ X-ray tube (monochromatic), the radiation of which does not cause fluorescence emission from the sample at a tube voltage of U = 3U_0_ (U_0_ is the exciting voltage), since the intensity of diffraction maxima depends on the kinematic scheme of the diffractometer (for example, the mathematical formulas for the Lorentz factor have different forms).

The states of the surface and near-surface layers of oxides were studied using infrared (IR) spectroscopy and X-ray photoelectron spectroscopy (XPS). The spectra were obtained using an Agilent Cary 630 IR spectrophotometer (made in the Santa Clara, CA, USA) in the frequency range of 1100–4000 cm^−1^ and a Nikolet 6700 (Thermo Fisher Scientific, Waltham, MA, USA) in the frequency range of 500–4000 cm^−1^ with an ATR (attenuated flat internal reflection) attachment. XPS was used to determine the oxidation states of the atoms in the oxide. The measurements were performed using a PHI5000 Versa Probe-II XPS microprobe (ULVAC-PHI) with a monochromatic Al Kα source (*hν* = 1486.6 eV, 15 kV) (made in the Chanhassen, MN, USA). A dual-beam device was used to neutralize the charge that occurs during the analysis of dielectric samples. Background modeling and subtraction, peak fitting, and quantification of elemental oxidation state contents were performed using Casa XPS software version 2.3.16.

The parameters of the porous structure and the specific surface area of the oxides were evaluated using an automatic TriStar II gas adsorption analyzer (made in the Micromeritics, Norcross, GA, USA). The specific surface area was measured using the BET (Brunauer–Emmett–Teller) method. The volume and pore size were determined using the BJH (Barrett–Joyner–Halenda) model, based on data from adsorption and desorption isotherms at a relative pressure of *P*/*P*_0_ = 0.99. The accuracy of the method was 5–10% for the relative pressures from 0.05 to 0.35.

The morphology of the powder surface was studied using a Hitachi TM-3000 scanning electron microscope (made in Tokyo, Japan) with an accelerating voltage of 15 kV under conditions where sample charging was removed (electron gun of 5 × 10^2^ Pa, sample chamber of 30–50 Pa). The X-ray spectrometer QUANTAX-70 (Bruker, Billerica, Massachusetts, USA) was used to obtain the elemental compositions from the EDX (energy-dispersive X-ray) spectra. The diffuse reflection spectra of oxides were recorded using a UV-2501 PC Shimadzu spectrophotometer with an ISP-250A diffuse reflection prefix (made in Kyoto, Japan). The spectra of the samples were taken relative to BaSO_4_ in the wavelength range from 190 to 900 nm. The final spectra were represented as Kubelka–Munk function.

Methyl orange (MO) was used as a model organic contaminant for investigating the photocatalytic activities of the prepared samples. First, 120 mg of sample was added to a 42 mL water solution of dye with a concentration of 13 mg L^−1^ in a quartz glass. The mixture was then stirred for 1 h in the dark in order to reach adsorption–desorption equilibrium. After that, it was irradiated under an ultraviolet iodine excilamp (model I_2__BD_P) with λ_max_ = 342 nm for 60 min. Throughout the experiment mixture was continuous stirring. At regular time intervals (10 min), aliquots were taken from the mixture and centrifuged. The dye concentration was determined on a PE-5400 UV spectrophotometer (ECROSKHIM Co., Ltd., Saint-Petersburg, Russia) by measuring the intensity of maximum absorption of MO at 461 nm using the Kin5400 kinetic analysis software version 2.0.

Additionally, an experiment was conducted to confirm that tin oxide is involved in the photocatalytic degradation of an organic dye. The experiment was performed under similar conditions without a photocatalyst. As a comparison sample in this work, we used the industrial photocatalyst P25 (TiO_2_).

All experiments on photocatalytic activity were repeated at least 6 times. After that, the results were averaged (dark sorption, degree of MO conversion under UV irradiation, photodegradation rate constant) to determine the confidence interval.

## 4. Conclusions

Polycrystalline powders of tin(II) oxide with different crystallographic textures were obtained by MW and HTMW heating of tin oxohydroxide suspensions in ammonia and carbonate media. The oxide formed at the interface between the Sn_6_O_4_(OH)_4_ solid phase and the alkaline dispersion medium, where the hydroxyl groups in the medium initiate the chemical decomposition of Sn_6_O_4_(OH)_4_ solid. The crystallographic texture of polycrystalline tin(II) oxide can be changed as a result of the recrystallization of crystallites under pressure and by increasing the holding time of SnO suspensions in different dispersion media:

- Recrystallization of SnO crystallites in the ammonia solution occurs due to the growth of crystallites in the (*h0l*) plane, and in the presence of sodium carbonate in the (*00l*) plane;
- The pressure in the reaction system promotes recrystallization, leading to an increase in the number of SnO crystallites that are oriented in the (*00l*) plane, regardless of the composition of the dispersion medium.

The optical properties of polycrystalline oxide SnO depend on its crystallographic texture. The value of the band gap of SnO powder increases from 3.0 eV to 3.6 eV with an increase in the content of crystallites oriented in the (*00l*) plane, from 17% to 49%. This increase in the number of such crystallites is associated with the formation of fewer electron-deficient tin(II) ions located in various oxygen environments on the surface of the tin(II) oxide. These dependencies can be explained using band theory. Different electron states in a solid, depending on the oxygen atom’s environment by tin atoms, lead to changes in electron density distribution in energy bands. This assumption about a change in the optical band gap of substances depending on the direction of crystal growth is confirmed by both theoretical calculations within the framework of the density functional theory, calculated for some oxide structures [25,57,58], and experimental data [28].

The photocatalytic and sorption anisotropy of oxides is related to the geometric and energetic accessibility of tin atoms on the surface and near-surface layers of the solid, which is also determined by the crystallographic texture of polycrystals. Polycrystalline SnO powders with a repeatability coefficient P_(*00l*)_ ranging from 17 to 40% exhibit photocatalytic activity in the photodegradation reaction of the azo dye (methyl orange). They are characterized by a dark sorption capacity for MO ranging from 35 to 49 wt. %. The degree of MO conversion is at least 96% after 1 h of photocatalytic treatment. The rate of decomposition of MO using SnO photocatalysts is 2–4 times higher than the rate of decomposition for the industrial photocatalyst Evonic Degussa P25.

The established patterns of synthesis of polycrystalline tin(II) oxide with different crystallographic textures expand our understanding of the production of optically active polycrystals crystallizing in the middle syngony with specified properties.

## Figures and Tables

**Table 1:** Properties of SnO.

Form of Agglomerates	Nanoplate Size, Length/Width/Thickness, nm	S, m^2^/g	ΔE, eV	Dye */k_υ_, 10^−2^ min^−1^	Reference
Flower	800/400/25	7.59	2.59	MO/0.996	[12]
Square	100–200/–/–	–	2.99	MB/1.126 RdB/0.593	[14]
Sphere	10–50/–/–	–	1.12	–	[19]
Sphere	22.6/–/– 21.7/–/– 19.3/–/–	73.46 108.44 157.45	3.70 4.00 4.20	–	[16]
Flower	10–30/–/–	52.90	2.65	MO/–	[17]
Flower	–/–/48	–	2.60	KG/0.390	[1]
Flower	–/–/20–30	–	2.75	MO/–	[18]
Flower	500/–/–	–	–	MO/0.013	[20]
Flower	–	–	1.46	–	[11]

* MO: methylene orange, MB: methylene blue, RdB: rhodamine B, KG: acid blue.

**Table 2:** Quantitative EDX results of polycrystalline SnO samples obtained under microwave exposure.

The Composition of Suspension Medium	Time of MW Treatment							
	5 min		7 min		10 min		15 min	
	Element, ±5 at. %							
	Sn	O	Sn	O	Sn	O	Sn	O
NH_4_Cl–NH_3_	51.6	48.4	51.2	48.8	50.9	49.1	51.1	48.9
NaCl–NaOH– Na_2_CO_3_	50.9	49.1	51.5	48.5	50.3	49.7	50.9	49.1

**Table 3:** Designations, Lotgering coefficients (*f*_(*hkl*)_), and repeatability factors (P_(*00l*)_) of the prepared polycrystalline SnO powders.

Group A		Group B	
Sample, A_P*00l*_	Lotgering coefficients *f* (±0.002) and repeatability factor P (±0.2%)	Sample, Б_P*00l*_	Lotgering coefficients *f* (±0.002) and repeatability factor P (±0.2%)
1A_17.1_	*f*_(*h0l*)_ = 0.082; *f*_(*00l*)_ = – P_(*00l*)_ = 17.1%	1B_27.6_	*f*_(*h0l*)_ = –; *f*_(*00l*)_ = 0.122 P_(*00l*)_ = 27.6%
2A_29.8_	*f*_(*h0l*)_ = 0.330; *f*_(*00l*)_ = – P_(*00l*)_ = 29.8%	2B_39.1_	*f*_(*h0l*)_ = –; *f*_(*00l*)_ = 0.167 P_(*00l*)_ = 39.1%
3A_33.1_	*f*_(*h0l*)_ = 0.382; *f*_(*00l*)_ = – P_(*00l*)_ = 33.1%	3B_48.8_	*f*_(*h0l*)_ = –; *f*_(*00l*)_ = 0.311 P_(*00l*)_ = 48.8%

**Table 4:** The results of dark sorption and photodegradation of MO (α—degree of MO conversion under UV irradiation (λ = 342 nm) and k_ν_—photodegradation rate constant) in the presence of the polycrystalline SnO powders and Evonic Degussa P25.

Photocatalyst	Sorption of MO, ±1 wt. %	α, ±0.5 wt. %	k_ν_·10^−2^, ±0.1 min^−1^
1A_17.1_	49	95.0	1.3
2A_29.8_	36	96.2	2.8
3A_33.1_	35	96.0	2.7
1B_27.6_	44	65.0	0.9
2B_39.1_	46	54.1	0.5
3B_48.8_	4	–	–
P25 (TiO_2_)	27	43.9	0.4

## Data Availability

Data will be made available on request.
